# Drug Abuse Educational Program for Saudi University Students: A Pre-test and Post-test Design

**DOI:** 10.7759/cureus.56079

**Published:** 2024-03-13

**Authors:** Safah Faqih, Muna Alharbi

**Affiliations:** 1 Nursing, Umm Al-Qura University, Mecca, SAU

**Keywords:** substance abuse program, educational programs, awareness, university students, drug abuse

## Abstract

Background and objective

Designing a consistent preventive drug abuse program and evaluating the educational needs of diverse target groups, school curricula, and new instructional materials customized to a country's socioeconomic and cultural characteristics should be used. This research aims to assess the influence of an educational program on university students' awareness of drug abuse.

Methodology

A cross-sectional study using a pre-test questionnaire with predetermined questions was conducted with 102 participants. The educational program about drug abuse prevention measures was subsequently implemented. After the program was completed, a post-test was administered to the students, and the results were compared to the pre-test results. The data were collected from male and female Saudi students at a university in the western area of Saudi Arabia.

Results

The pre-test findings indicate that the students had already received some information on drug use and abuse, but the post-test results show that their awareness rose as a result of the drug abuse educational program.

Conclusion

Drug abuse is a major problem all across the world, including in Saudi Arabia. However, educational programs will help to increase knowledge and awareness of this issue.

## Introduction

The biological and psychosocial problem of drug and substance abuse poses a significant threat to public health in many nations and to human society worldwide [1-3]. Alcohol, opiates, cocaine, cannabinoids, and amphetamines are the most frequently misused drugs [4]. Youth populations are particularly at risk in developing nations where the circumstances may be favorable to substance use [3]. Students engage in substance use for various reasons, despite the consequences [5]. The factors of sex, age, peer pressure, family substance abuse, personal pleasure, and low academic achievement have all been linked to student substance use [5-7].

In addition to reporting novice use, studies have found that students who were previously exposed to substance use increased the frequency of their substance use after entering the university environment [8,9]. In a cross-sectional study that was conducted in nine Asian countries, it was reported that the overall prevalence of binge drinking among university students was less than 2% in Indonesia and Malaysia, 39.1% in Thailand, and 55% in Laos [10]. The religious affiliations of students may account for these variations. Alcohol consumption is more restricted in Muslim nations like Indonesia and Malaysia than in Buddhist nations such as Thailand and Laos, where the general population consumes more alcohol [10].

Among individuals in Kenya between the ages of 15 and 24 years, the national prevalence of lifetime substance use is 37.1%, while the prevalence of current substance use is 19.8% [11]. The higher trends in the current use of cannabis and alcohol among college students may be explained by the normalization of substance use behavior as well as the aggressive marketing of alcohol and other drugs to college students on digital platforms and social media, such as Facebook and WhatsApp, of which college students are frequent users [12].

In the Hohoe municipality of Ghana, tertiary students have a high lifetime prevalence of alcohol consumption, which has a number of physical, psychological, and financial consequences. Higher education institutions should provide more alcohol-related education, and counseling departments should have access to appropriate assessment tools for evaluating and assisting students who are already abusing alcohol [13].

Research has made it abundantly clear that the issue of substance abuse among college students is growing in importance. In a study at Wolaita Sodo University, 28.6% of students reported using drugs or alcohol. Alcohol was the most frequently used substance, followed by cigarettes and tobacco products [7]. These findings indicate a lower prevalence than that identified by a study in northern Ethiopia [5], but they are in line with the results of a study conducted in southern Iran [14].

Researchers in Ethiopia have reported that alcohol was consumed by 24.7% of students at various higher education institutions, 21.6% of medical students at Addis Ababa University, 20% of students at Haramaya University, and 21.7% of college students in southern Ethiopia [15,16]. However, this prevalence is lower than that reported in a study with Axum University students, where alcohol consumption was 32.8% higher. In addition, the prevalence is significantly lower than that recorded in other studies of university students in Turkey and Trinidad and Tobago, where students’ six-month alcohol consumption was 37.9% and 70%, respectively [17-19]. Cultural and socioeconomic differences could explain these discrepancies [7].

Smoking is a more significant problem among Saudi college students compared to the wider Saudi population as well as neighboring nations. In one study, the pooled estimate of tobacco smoking among college students in the Kingdom of Saudi Arabia was 17%, which is 5% higher than the average prevalence of current daily smokers aged 15 to 25 years in Saudi Arabia [20]. Furthermore, a nationally representative study has revealed that the rate of smoking among Saudi college students is higher than that of a similar age group [21].

To respond to the high prevalence of substance use, which has been associated with poor academic performance, universities must effectively control substance use and implement youth-friendly activities to influence behavioral change in students. For instance, the cumulative grade point average of female students can be improved by encouraging and empowering them to actively participate in teaching and learning activities [7]. The attitudes of high school students toward drug abuse and addiction can also be significantly changed through life skills training and lecture- and video-based educational methods of prevention programs. In regard to changing attitudes toward drugs and addiction, research has shown that a method using video clips was more effective with male students, whereas a method focused on group discussion achieved better results among female students [22].

School curricula and innovative educational materials that are tailored to a country’s socioeconomic and cultural conditions should be employed to develop a consistent preventive drug abuse program and determine the educational needs of various target groups [22]. For that purpose, this research aims to assess the influence of an educational program on university students' awareness of drug abuse.

## Materials and methods

Research design, sampling, and setting

This study applied a cross-sectional research design using pre-test and post-test questionnaires. The research sample was comprised of male and female Saudi students at a selected university in the western region of Saudi Arabia.

Data collection

For the data collection, a pre-test questionnaire with predetermined questions was administered to 102 participants who attended the educational session. The questionnaire was formulated based on the validated survey instrument created by Geramian et al. in 2014 [4]. The questionnaire was originally written in English and then translated into Arabic for the final version. The questions were translated through forward and backward translation techniques. A group of professionals tested the translated survey instrument for face and content validity. It was verified by two academics and two students who were all native Arabic speakers with substantial experience in creating and validating survey instruments. The information was revised in response to their suggestions.

The final version of the survey instrument consisted of 16 questions, divided into three sections. The first section (four items) concerned the demographic characteristics of participants, including their age, family size, and father’s educational level and occupation. The second section (two items) evaluated students’ smoking status and history of drug abuse education using a binary scale (yes/no). The third section (one item) contained a multiple-choice question to be answered with a five-point Likert scale (1 = not important, 2 = slightly important, 3 = fairly important, 4 = important, 5 = very important).

The researchers implemented an educational session about drug abuse prevention measures. A post-test using the same questionnaire was administered to the students after the program to enable a comparison between their pre-test and post-test results.

Data analysis

The data were collected, reviewed, and then analyzed with SPSS Statistics version 21 (IBM Corp., Released 2012. IBM SPSS Statistics for Windows, Version 21.0. Armonk, NY: IBM Corp.). The analysis used two-tailed statistical methods with an alpha level of 0.05. A p-value of less than or equal to 0.05 was considered statistically significant. The overall knowledge and awareness level regarding addiction was assessed by summing up discrete scores for different correct awareness items. The awareness level was categorized as poor if the student’s score was less than 60% of the overall score and good if the score was 60% or more of the overall score. Descriptive analysis was performed by prescribing the frequency distribution and percentage for study variables, including the students’ personal data and family data and whether they had received information about addiction. Furthermore, students’ awareness of addiction before and after the intervention was compared using a marginal homogeneity test for related samples, and a graph was made for overall awareness. Students’ mean rankings for factors associated with addiction were compared. Their attitudes toward the effects of addiction and their ideas about drug use for addiction were also compared using the McNemar test.

Ethical considerations

Approval for this research was obtained from the Institutional Review Board of Umm Al-Qura University (approval number: HAPO-02-K-012-2023-5-1630). This study adopted measures to protect the privacy and autonomy of participants. No names or other personal identifying information were recorded during the data collection process. By eliminating any components that could be used to identify participants, the data were anonymized. Codes were assigned to the data to avoid disclosing any identifying information in the research paperwork.

## Results

A total of 102 university students were included in the analysis. Sixty-seven (65.7%) were students at health colleges, while 35 (34.3%) were students at non-health colleges. Their ages ranged from 18 to 27 years old, with a mean age of 22.1 ± 3.5 years old. Sixty-six (64.7%) of the students were female. Forty-two (41.2%) students had a father who was a university graduate, 40 (39.2%) had a mother with a secondary level of education, and 36 (35.3%) had a mother who had graduated from a university. Twenty-eight (27.5%) students had families with up to five members, while 35 (34.3%) had families with eight or more members. Fifty-eight (56.9%) students had a father who was retired or not working, while 35 (34.3%) students indicated that their father was an employee. Eighty-nine (87.3%) students reported that they had previously received information about drug use (Table 1).

**Table 1:** Socio-demographic data of participating university students in Saudi Arabia (n=102)

Socio-demographics	No	%
Age in years		
18-20	19	18.6%
21-22	49	48.0%
23+	34	33.3%
Gender		
Male	36	35.3%
Female	66	64.7%
College		
Non-health college	35	34.3%
Health college	67	65.7%
Father’s education		
Below secondary	34	33.3%
Secondary	26	25.5%
University/above	42	41.2%
Mother’s education		
Below secondary	40	39.2%
Secondary	26	25.5%
University/above	36	35.3%
Family size		
<5	28	27.5%
5-7	39	38.2%
8+	35	34.3%
Father’s work		
Not working/retired	58	56.9%
Employee	35	34.3%
Freelancer	9	8.8%
Have you received any information about drug use?		
Yes	89	87.3%
No	13	12.7%

Table 2 presents data on the university students’ awareness of drugs and addiction before and after the intervention (n=102). All of the differences from pre- to post-intervention were statistically significant (p<0.01).

**Table 2:** University students’ awareness of drugs and addiction before and after intervention (n=102) P: test of marginal homogeneity, * p<0.05 (significant)

Awareness items		Phase				p-value
		Pre-test		Post-test		
		No	%	No	%	
Methamphetamine effects	Tachycardia	71	70.3%	101	99.0%	0.001*
	Tooth damage	64	63.4%	100	98.0%	
	Violent behaviors	92	91.1%	100	98.0%	
	Hypertension with stroke	53	52.5%	100	98.0%	
	Excessive talking	32	31.7%	101	99.0%	
	Paranoia	70	69.3%	101	99.0%	
	Carelessness	42	41.6%	102	100.0%	
	Low body temperature	17	16.8%	6	5.9%	
	Feeling comfort	11	10.9%	0	0.0%	
	Weight gain	12	11.9%	0	0.0%	
Signs of an addicted person	Sudden change in lifestyle	92	90.2%	102	100.0%	0.001*
	Extravagance and increased demand for money	87	85.3%	102	100.0%	
	Tendency to withdraw and loneliness	74	72.5%	102	100.0%	
	Leaving home for prolonged periods of time and staying out late at night	74	72.5%	101	99.0%	
	Dealing confidentiality regarding particularities	35	34.3%	99	97.1%	
	Attention to appearance	2	2.0%	1	1.0%	
	More social relations	53	52.0%	100	98.0%	
	Improved academic performance	6	5.9%	0	0.0%	
	Weight gain	11	10.8%	1	1.0%	
Symptoms associated with steroids	Euphoria and irritation	96	94.1%	101	99.0%	0.001*
	Paranoia	60	58.8%	101	99.0%	
	Nasal congestion and damage to the nasal mucosa	41	40.2%	101	99.0%	
	Decreased heart rate, blood pressure, and temperature	35	34.3%	5	4.9%	
	Weight gain	16	15.7%	0	0.0%	
Long-term alcohol-associated problems	Increased risk of heart disease and stroke	79	77.5%	100	98.0%	0.001*
	Harm to the brain and nervous system	84	82.4%	101	99.0%	
	Gastrointestinal disease disorders	71	69.6%	102	100.0%	
	Severe damage to the liver	82	80.4%	101	99.0%	
	Nausea	52	51.0%	2	2.0%	
Short-term alcohol-associated problems	Blurred vision	61	59.8%	101	99.0%	0.001*
	Vomiting	79	77.5%	102	100.0%	
	Headache	82	80.4%	98	96.1%	
	Slurred speech	60	58.8%	101	99.0%	
The most common drugs in the Kingdom of Saudi Arabia	Hashish	64	62.7%	102	100.0%	0.001*
	Captagon pills	44	43.1%	99	97.1%	
	Shabu	87	85.3%	101	99.0%	
	Heroin	28	27.5%	2	2.0%	

Prior to the intervention, the most commonly known methamphetamine effects were violent behaviors (91.1% vs. 98% post-intervention), tachycardia (70.3% vs. 99%), paranoia (69.3% vs. 99%), and tooth damage (63.4% vs. 98%). The most commonly known signs of an addicted person were a sudden change in lifestyle (90.2% vs. 100%), extravagance and increased demand for money (85.3% vs. 100%), a tendency to withdraw and loneliness (72.5% vs. 100%), and leaving home for prolonged periods of time and staying out late at night (72.5% vs. 100%). The most reported symptoms associated with steroids were euphoria and irritation (94.1% vs. 99%), paranoia (58.8% vs. 99%), and nasal congestion and damage to the nasal mucosa (40.2% vs. 99%). The most frequently identified long-term alcohol-associated problems were harm to the brain and nervous system (82.4% vs. 99%), severe damage to the liver (80.4% vs. 99%), increased risk of heart disease and stroke (77.5% vs. 98%), and GIT disorders (69.6% vs. 100%). The most documented short-term alcohol-associated problems were headaches (80.4% vs. 96.1%), vomiting (77.5% vs. 100%), blurred vision (59.8% vs. 99%), and slurred speech (58.8% vs. 99%).

Regarding the most common drugs, Shabu was identified by 85.3% of the students before the intervention and by 99% after the intervention. Hashish was reported by 62.7% of the students before the intervention and by 100% after the intervention. Finally, Captagon pills were known by 43.1% of students before the intervention, compared to 97.1% after the intervention.

**Figure 1:**
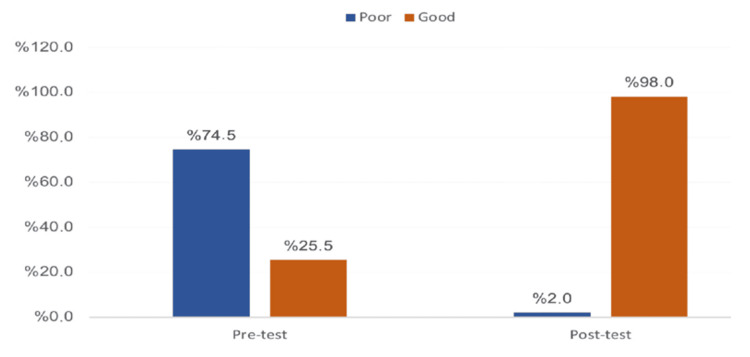
Overall awareness level of the university students in Saudi Arabia regarding drugs and addiction

Figure 1 depicts the students’ overall awareness level regarding drugs and addiction. Twenty-six (25.5%) of the participants had an overall good awareness before the intervention lectures. This figure increased significantly to 100 (98%) after the intervention, with recorded statistical significance (p=0.001).

Table 3 presents the factors that contribute to drug addiction reported by university students in Saudi Arabia. Before the intervention, the most highly ranked factors were the presence of an addicted person in a residential or educational setting (4/5), the pursuit of pleasure (3.9/5), parental divorce (3.8/5), mental disorder (3.8/5), and family conflicts (3.8/5). After the intervention, the highest-ranked factors included the presence of an addicted person in a residential or educational setting (5/5), the presence of an addict in the family (4.9/5), the ability to obtain drugs (4.4/5), the low cost of drugs (4.3/5), and the inability to access counseling centers (4.2/5).

**Table 3:** Factors that contribute to drug addiction as reported by the university students in Saudi Arabia

Factors	Phase			
	Pre-test		Post-test	
	Mean	SD	Mean	SD
Teen curiosity	3.8	1.1	3.3	0.7
The pursuit of pleasure	3.9	1.2	3.4	0.7
Physical diseases	2.5	1.4	3.2	0.7
Mental disorder	3.8	1.2	3.2	0.8
Lack of knowledge of medication complications	3.3	1.4	3.4	0.8
Positive perception of drugs	2.9	1.6	1.8	1.0
Low self-confidence	3.4	1.4	2.2	1.0
Eliminating shyness	2.6	1.4	2.0	1.0
Parental divorce	3.8	1.3	3.5	0.8
Lack of entertainment facilities	3.0	1.3	3.1	0.9
Inability to solve routine problems	3.3	1.4	3.5	0.7
Large family	2.7	1.5	3.4	0.8
Having strict parents	3.0	1.5	4.0	0.6
The presence of an addict in the family	3.7	1.4	4.9	0.4
Family conflicts	3.8	1.2	4.2	0.6
Ability to obtain drugs	3.4	1.3	4.4	0.7
Inability to access counseling centers	2.9	1.3	4.2	0.7
Low cost of drugs	2.5	1.5	4.3	0.7
Having free time	3.6	1.3	3.9	0.7
The presence of an addicted person in a residential or educational setting	4.0	1.2	5.0	0.3

Table 4 shows the participants’ perceptions and attitudes toward addiction. Before the intervention, 80.4% agreed that addiction causes temporary euphoria or happiness, while 80.2% agreed with this after the intervention (p=0.07). Before the intervention, 3.9% agreed that addiction can improve memory and learning, while 1% agreed with this after the intervention (p=0.009). Before the intervention, 77.5% agreed that addiction causes depression; this increased to 97.1% after the intervention (p=0.001). While 8.8% agreed that addiction could improve some physical diseases before the intervention, no students agreed with this after the intervention (p=0.001). The assertion that addiction increases self-confidence was agreed with by 7.8% of participants before the intervention but only 1% after the intervention. Finally, 11.8% agreed that addiction increases acceptance by friends before the intervention, but no student agreed with this after the intervention (p=0.001).

**Table 4:** Perceptions and attitudes toward addiction among university students in Saudi Arabia P: McNemar test for related samples, * p<0.05 (significant)

Attitude	Phase				p-value
	Pre-test		Post-test		
	No	%	No	%	
Temporary euphoria or happiness					0.007*
Disagree	12	11.8%	20	19.8%	
Not sure	8	7.8%	0	0.0%	
Agree	82	80.4%	81	80.2%	
Improve memory and learning ability					0.009*
Disagree	91	89.2%	101	99.0%	
Not sure	7	6.9%	0	0.0%	
Agree	4	3.9%	1	1.0%	
Depression					0.001*
Disagree	6	5.9%	3	2.9%	
Not sure	17	16.7%	0	0.0%	
Agree	79	77.5%	99	97.1%	
Improve some physical diseases					0.001*
Disagree	80	78.4%	101	99.0%	
Not sure	13	12.7%	1	1.0%	
Agree	9	8.8%	0	0.0%	
Increase self-confidence					0.001*
Disagree	73	71.6%	101	99.0%	
Not sure	21	20.6%	0	0.0%	
Agree	8	7.8%	1	1.0%	
Increase acceptance by friends					0.001*
Disagree	76	74.5%	101	99.0%	
Not sure	14	13.7%	1	1.0%	
Agree	12	11.8%	0	0.0%	

**Figure 2:**
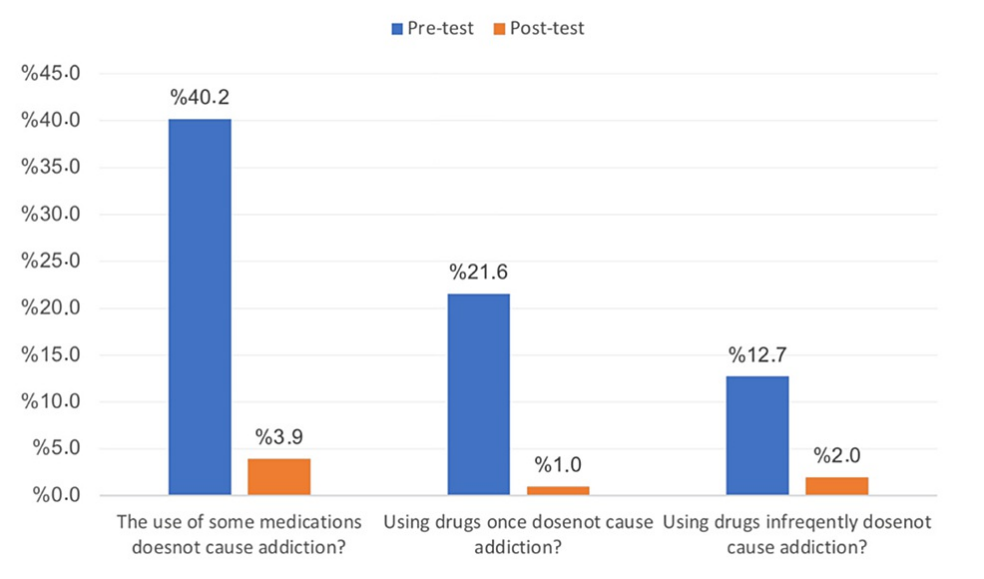
University students’ ideas about the use of drugs in addiction before and after the intervention

Figure 2 illustrates the university students’ ideas about the use of drugs in addiction before and after the intervention. Before the intervention, 40.2% agreed that the use of some medications does not cause addiction; after the intervention, only 3.9% agreed with this. Before the intervention, 21.6% agreed that using drugs once did not cause addiction. This figure fell to 1% after the intervention. Finally, before the intervention, 12.7% agreed that using drugs infrequently does not cause addiction, but only 2% agreed with this after the intervention. These results showed statistical significance (p<0.05).

## Discussion

This study aims to evaluate the impact of an educational program on increasing awareness of the dangers of drug abuse among students at a selected university in Saudi Arabia. Of the 102 total participants, 67 (65.7%) were health college students, 35 (34.3%) were students at non-health colleges, 36 (35.3%) were male, and 66 (64.7%) were female. The average age of the study participants was between 21 and 22. A questionnaire was administered to assess the level of information before and after the educational sessions and examine students’ responses to the educational program. It was found that 89 (78.3%) students had previously received information about drug use.

The results indicate that the educational program increased awareness among both male and female students, as their awareness levels were significantly higher after the educational sessions. While there were clearly errors in answering the various questions on the pre-test, many students agreed on their answers to the post-test. These results are similar to those of a previous study that assessed drug use among high school students in Isfahan Province, Iran, which noted that structured continuing programs can improve students’ knowledge of and attitudes toward drug use [4].

On the post-test, the participants indicated that the most commonly used drugs in the Kingdom of Saudi Arabia are Shabu (102; 100%), hashish (101; 99%), and Captagon pills (99; 97.1%). In contrast, according to Global Statistics on Alcohol, Tobacco, and Illicit Drug Use: Status Report 2017, the high-income region of North America scored the highest dependence rates for cannabis, opiates, and cocaine [23].

In the present study, a large number of students agreed on the post-test that major factors contributing to drug addiction in individuals are the presence of an addict in one’s place of residence, the presence of an addict in the family, access to drugs, low drug prices, family problems, an inability to access counseling centers, and having strict parents Similarly, in another study, participants mentioned that the top reasons for drug use were an offer from friends (132 respondents; 41.7%), seeking pleasure (129 respondents; 40.8%), parental divorce (126 respondents; 39.8%), obtaining drugs (125 respondents; 39.5%), family issues (110 respondents; 34.8%), addiction of a family member (66 respondents; 20.8%), and curiosity (101 respondents; 31.9%). Transient euphoria (75.9%), followed by depression (62.3%), were considered the most prevalent physical or psychological changes that occur after drug use (p=0.0001) [24].

A cross-sectional study among undergraduates at selected southwestern universities in Nigeria has listed factors that influence drug consumption, according to the respondents. The most common reasons included “feeling euphoric” (81.3%), “to help you when you are feeling down or stressed” (65%), “forget your problems” (65%), and “to cheer you up when you are in a bad mood” (64.5%). A total of 59.8% (n=239) of the participants chose “not to feel left out” [25].

The results of the present study evidence that family-related factors, such as parental divorce, family problems, the presence of an addict in the family, and having strict parents, are influencing factors that contribute to drug use. Likewise, in a study of recreational drug use among Nigerian university students, family and domestic factors were identified as important predictors of recreational drug use. While being in a nuclear family was associated with a lower likelihood of drug use in the unadjusted model, the effect size was no longer significant after controlling for demographic factors. In the unadjusted regression analysis, living with a father protected against drug use, but the effect size decreased and was no longer significant after adjusting for significant covariates [26].

With regard to the expected effects of taking narcotic substances, the post-test results indicate that 81 students (80.2%) agreed that drugs can cause euphoria and transient happiness, while 99 students (97.1%) agreed that they can cause depression. Similarly, in a cross-sectional study with healthcare students in Saudi Arabia regarding the immediate effects of ingesting an offending or illicit substance or drug (during a number of hours), the majority of participants (240; 75.9%) agreed that transient euphoria occurs after the use of illicit drugs, and more than half of the 197 students (62.3%) agreed that depression can be caused by the use of drugs or illegal substances [24].

The post-test results in this study contradict the results of another study, where 101 students (99%) objected to the possibility of drug use improving some physical diseases, and the same number agreed that drugs do not improve memory or the ability to learn. In yet another study, nearly half of the 145 participants (45.9%) reported improvement in some physical ailments (pain and distress), followed by increased self-confidence [24].

In this study, students were also asked about substance use that may not cause addiction. On the pre-test, 40.2% of the participants agreed that using certain drugs does not cause addiction, but only 3.9% agreed with this on the post-test. In addition, 21.6% of the participants agreed on the pre-test that using drugs only once does not cause addiction, but only 1% agreed with this on the post-test. The agreement that using drugs infrequently does not cause addiction fell from 12.7% on the pre-test to 2% on the post-test. In another study, 212 students (67.1%) agreed that it is possible to become addicted (physically and/or psychologically) to drugs or illicit substances after one exposure, and 186 (58.9%) disagreed that occasional use of illegal drugs is acceptable. However, 146 (46.2%) students agreed that certain drugs, such as hashish, are not addictive [24].

This study has highlighted important statistics regarding awareness of drug abuse among university students and how educational programs can positively influence their awareness levels. Notably, the study has some limitations to consider. First, since the data were collected in a single setting, the results may not be generalizable to other contexts. Second, the study contains some sample bias, as most of the participants were from health fields, and non-health students may have different results.

## Conclusions

Drug abuse is a significant issue around the world, including in Saudi Arabia. However, educational programs offer an effective way to enhance knowledge and awareness of this problem. As a significant finding of this study, the pre-test results indicate that the students had already received some information about drug use and abuse, yet the post-test results reveal that their awareness nonetheless increased as a result of the drug abuse educational program.

An educational program of this type should provide an overview of drug abuse, including the types of drugs that are commonly abused, the effects of drug abuse on physical and mental health, and the prevalence of drug abuse. Moreover, it should educate students about the risk factors for drug abuse, such as peer pressure, stress, and mental health issues, as well as the warning signs of drug abuse in themselves and others. It should also inform them about prevention strategies, including healthy coping mechanisms, stress management techniques, social support networks, and available treatment options for drug abuse, which range from counseling and support groups to medication-assisted treatment. Finally, it should deliver information about accessible resources, such as hotlines, support groups, and treatment centers, for individuals who are struggling with drug abuse. It is important to note that the effectiveness of a drug abuse educational program depends on various factors, such as the quality of the program, the target audience, and the delivery method. Therefore, it is essential to evaluate a program’s effectiveness and make the necessary adjustments to ensure its success.
